# University scientists’ willingness to participate in public engagement: A concept explication

**DOI:** 10.1371/journal.pone.0337189

**Published:** 2025-11-25

**Authors:** Becca Beets, Mikhaila N. Calice, Lindsey Middleton, Dominique Brossard, Dietram A. Scheufele, Luye Bao, Todd P. Newman, Jo Handelsman

**Affiliations:** 1 Department of Communication, University of Maryland, College Park, Maryland, United States of America; 2 Morgridge Institute for Research, Madison, Wisconsin, United States of America; 3 Department of Life Sciences Communication, University of Wisconsin-Madison, Madison, Wisconsin, United States of America; 4 Peking University HSBC Business School, Shenzhen, China; 5 Wisconsin Institute for Discovery, University of Wisconsin-Madison, Madison, Wisconsin, United States of America; Instituto Tecnologico Autonomo de Mexico, MEXICO

## Abstract

Public engagement is increasingly recognized as a critical responsibility of the scientific community. Scientists in academic settings are well positioned to lead these efforts, but they are not always willing or able to participate in engagement. Public engagement can encompass a range of activities that may require different resources and skills, and which may have different outcomes for both scientists and non-scientists. Therefore, understanding which activities scientists are willing to participate in is critical for supporting their engagement efforts at the institutional level. Using survey data from a case study of science faculty at a large land-grant university in the United States, we conduct a systematic concept explication to better understand the dimensions of public engagement activities that scientists are willing to participate in. Based on thirteen different activities, we define and analyze the reliability of five dimensions of engagement: public scholarship, educational activities, direct engagement with public audiences, stakeholder-focused collaboration, and industry engagement. We also examine the validity of these five dimensions and how factors including institutional culture and norms, professional status, and attitudes towards engagement relate to scientists’ willingness to participate in engagement. Our results provide a robust categorization of willingness to engage as a blueprint for future research in this space.

## Introduction

Public engagement is increasingly recognized as a critical responsibility of the scientific community [1–3]. This need has only grown more pressing as emerging areas of science and technology, such as artificial intelligence, raise a host of regulatory, political, and moral questions that require engagement from both scientists and non-scientists [4–6]. Scientists in academic settings are well positioned to lead these efforts, but they are not always willing or able to participate in engagement activities [7]. Academic institutions can take steps to support scientists’ engagement efforts [8,9], yet institutional barriers to academic scientists’ engagement persist [10]. Toward that end, some have called for what they consider an overdue culture change in academia to support public engagement efforts and public dialogue about science issues [11].

Public engagement is often used as an umbrella term [12] that comprises different activities [13–15], goals and objectives [4,7,16], and audiences [1], among other features. In our study, public engagement is defined broadly as “the act of seeking and facilitating the sharing and exchange of knowledge, perspectives, and preferences between or among groups who often have differences in expertise” [17]. This definition of engagement encompasses a range of potential activities and audiences, from giving public lectures, to writing op-eds, to working directly with stakeholders such as policymakers and agricultural communities. In this study, we investigate participation in science-related public engagement activities with a focus on university science faculty as experts. We acknowledge, however, that scientific expertise can come from different disciplines and perspectives at the intersections of science and society. Public engagement activities may rely on different resources, require different skills, and have different tangible outcomes for scientists and non-scientists [15]. Therefore, understanding which activities scientists are willing to participate in is critical for supporting their engagement efforts at the institutional level.

Researchers have used different approaches for measuring scientists’ past involvement and future willingness to participate in public engagement activities. For example, actual engagement behaviors have been assessed by quantifying the number of popular articles written by scientists over a particular period [e.g., 18], as well as by asking scientists to self-report their participation in engagement activities over the previous year [e.g., 19,20]. One of the challenges of using this approach is that scientists’ past participation in engagement may have been constrained by a lack of access to engagement opportunities or other resources. An alternative approach involves assessing scientists’ willingness to participate in future engagement activities. Researchers have measured scientists’ willingness to engage by asking how ‘willing’ they are to participate in different forms of engagement [20–23], whether they ‘intend’ [24] or ‘plan’ to engage [25], or how much time they would like to spend on engagement [26]. However, it is unclear whether existing typologies for public engagement [e.g., 13,14,27] reflect the range of activities that scientists are willing to participate in.

To address this gap in the research, the current study builds on a careful synthesis of the literature on public engagement as well as previous qualitative research to develop a reliable and valid measure of scientists’ willingness to participate in public engagement activities. Using survey data from a case study of science faculty at a large land-grant university in the United States (n = 528), we conduct a systematic concept explication to better understand the different dimensions of public engagement activities that university scientists are willing to participate in. Concept explication is an iterative process that involves both defining and operationalizing a concept of interest [28,29]. Concept definition and operationalization, or meaning analysis, is a deductive process that begins with the theory and involves describing the dimensions of the concept of interest, in this case willingness to participate in public engagement activities. The next step, empirical analysis, is an inductive process that begins with an analysis of data to measure the concept and further refine the theory [28,29]. In this study, our empirical analysis balances considerations between the reliability and the validity of the dimensions.

First, we define and analyze the reliability of five dimensions of public engagement activities derived from the literature: public scholarship, educational activities, direct engagement with public audiences, stakeholder-focused collaboration, and industry engagement (RQ1). Second, we examine the validity of these five dimensions and evaluate how they relate to measures of past participation in engagement (H1), institutional culture and norms (RQ2), professional position (RQ3), and attitudes towards engagement (RQ4).

**Categorizing the dimensions of public engagement activities.** Researchers have categorized public engagement activities in a variety of ways [for a typology of engagement activites, see: 13]. As one example of this categorization, researchers distinguish types of engagement based on the flow of information. For instance, information may flow one-way from scientists to a public audience (e.g., talking to the media) or two-ways in an exchange of information (e.g., participating in a community science event) [30]. Engagement activities can also be grouped by modality, such as communication, consultation, involvement, collaboration, and empowerment [4] or by the specific mechanism of engagement [13]. Other categorizations include distinctions based on the activity’s theme, purpose, or audience size [27] and the intensity of participation required by the engagement activity [14]. Given the many ways public engagement activities have been categorized, research across this space is not always able to be systematically compared. Based on the literature, and as synthesized below, we examine willingness to participate in thirteen public engagement activities, which we group into five dimensions. We elaborate on these dimensions and outline each of the thirteen corresponding activities, which are numbered 1–13 below.

Scientists often interact with media to communicate their research. Such types of communication are widely explored in public engagement research [15]. For example, research examining scientists’ engagement activities with the media has included (1) *publishing non-academic pieces*, such as op-eds or popular news articles, as well as (2) *participation in interviews with journalists*. Studies focusing on this type of engagement include the examination of nanoscientists’ contacts with media [25], as well as an exploration into scientists’ willingness to participate in media engagement [20]. In addition to interactions with popular media, engagement has been studied in terms of (3) *interacting with government bodies or officials*, such as by providing expert testimony [31] or meeting with policymakers [32]. These three activities, which involve interactions with the media and policymakers, have been previously grouped as a category of engagement activities reflecting scientists’ participation in “public debate and democratic decision-making” [19]. We refer to this dimension of engagement activities as **public scholarship**.

Another commonly analyzed public engagement activity involves participation in educational activities that often take place in educational institutions including both K-12 and higher education settings. Importantly, these types of activities are outside of the traditional teaching responsibilities that a scientist may have. For example, scientists may (4) *participate in science education efforts in K-12 environments* such as tutoring and mentoring children [33] or they may (5) *participate in science festivals* [34]. Scientists may also take part in less formal activities where, for instance, they (6) *interact with students outside of curricular activities* such as by mentoring students for research activities [10]. Prior research on scientists’ participation in these types of educational activities has explored how factors such as academic productivity influence scientists’ willingness to participate in engagement [35]. We refer to this dimension of engagement activities as **educational activities**.

Public engagement can also include various ways of directly engaging with audiences in both formal and informal settings. For instance, (7) *presenting research to the public, such as at a science café or formal lecture series* is a common form of engagement analyzed in the scholarly literature [32]. Another example of this type of ‘face-to-face’ engagement includes giving presentations and demonstrations [20]. Additionally, as our media ecology has evolved, researchers have examined scientists’ participation in online engagement with the public such as (8) *engaging on social media about research* [36,37]. Scientists may also engage in dialogue about research in informal settings by (9) *discussing research during everyday interactions with the public,* which may happen through un-scheduled face to face conversations with lay publics [38] or during “discussions, conversations, exchanges, or dialogues with the public” [15]. These types of engagement broadly represent approaches for disseminating research directly to public audiences across in-person and online settings. We refer to this dimension of engagement activities as **direct engagement with public audiences.**

There is increasing pressure on scientists to employ inclusive engagement and communication strategies in their research, especially with affected publics [39]. As such, public engagement has also been classified in terms of the levels of inclusion of different audiences into the research process. One example of (10) *including members of the public directly in the research process* is through participation in citizen science projects [40,41]. Another example of work with affected publics involves scientists’ (11) *work on extension-related activities* such as developing or participating in extension outreach programs at land-grant institutions [42]. In the United States, land-grant institutions were established through the Morrill Acts of 1862 and 1890, which allocated federal lands or funding to establish educational institutions [43]. These institutions have engagement-focused missions [44] and also include a Cooperative Extension Service [45]. Work through extension programs may involve disseminating practical research findings to community members as well as research that involves collaboration and input from key stakeholders [e.g., 46,47]. In line with this, faculty holding extension appointments have been shown to participate in engagement activities with stakeholders more frequently than faculty not holding extension appointments [48]. We refer to this dimension of engagement activities as **stakeholder-focused collaboration**.

Lastly, recent research has recognized industry as a key audience for engagement with science [15]. Research on academic-industry partnerships includes a focus on academic entrepreneurship and commercialization, which examines how university-industry relations impact scientists’ engagement with industry and the commercialization of research into products [for a review, see: 49,50]. The goals of university-industry collaborations can include both knowledge-focused collaboration to publish academic research, as well as property-focused collaboration aimed at the development of marketable products [51]. For many science disciplines, there is a research-to-market pipeline that includes (12) *translating research into marketable products* and (13) *collaborating with industry or professional stakeholders* on research. We refer to this dimension of engagement activities as **industry engagement**.

In sum, through our review of the literature we identified 13 engagement activities that we categorize into five dimensions of engagement: public scholarship, educational activities, direct engagement with public audiences, stakeholder-focused collaboration, and industry engagement. As a first step in our concept explication, we examine the reliability of each of these five dimensions, in other words, we assess how consistent the outcome of a measure is when it is repeated [28,52]. Thus, we ask:

RQ1. How *reliable* are the five dimensions of activities for willingness to participate in public engagement?

### Factors associated with scientists’ willingness to participate in engagement

Building on this research, as a second step in our concept explication, we examine the validity of our measure of willingness to participate in the five dimensions engagement activities. Validity refers to how accurately a measure reflects the concept of interest [28,52]. In this study, we specifically examine the construct validity of our dimensions, which entails comparisons with other variables that we would expect to be theoretically related [52,53]. To do this, we explore how willingness to participate in each of the dimensions of engagement activities relates to external factors representing institutional culture and norms, professional position, attitudes towards engagement, and past participation in engagement.

#### Institutional culture and norms.

The culture of an academic institution can be broadly understood as the assumptions and values that guide behavior in the workplace [54]. For any given institution, the culture reflects a systematic pattern of norms and attitudes within the organization and at the boundary with other organizations and the public [55]. In North America, academic institutions often have similar structures and hierarchies [56]. Thus, the culture and norms of academic institutions are important drivers of how academic scientists spend their time and what resources or engagement opportunities they have access to.

Institutional factors have been shown to affect perceptions about the behavioral norms for participating in engagement, which can impact scientists’ willingness to engage. However, findings on this are mixed. For example, one study found that perceptions of colleagues’ engagement behaviors was associated with scientists’ intentions to participate in public engagement, while perceptions about what others would approve or disapprove of regarding engagement were not [24]. Importantly, this study did not distinguish between types of engagement activities, and it measured intention rather than willingness. Another study—which distinguished between face-to-face, online, and media engagement activities—found that beliefs about colleagues’ engagement participation and perceptions about their approval of engagement were generally inconsistent predictors of willingness to engage [20].

A related line of research explores how perceptions of different institutional actors’ support for engagement affects the culture of engagement in university settings. For example, the perception that high-level administrators prioritize engagement at their university significantly predicted the engagement of science faculty in the United States [19]. These findings align with other work that suggests there are both top-down (e.g., from leadership) and bottom-up (e.g., from graduate students) influences on faculty engagement behaviors [10]. Additionally, scientists in the United States consistently perceive themselves to hold engagement in higher regard than their peers and their institutions [7,36]. For instance, one study found that scientists from a sample of U.S. institutions believed that they gave higher priority to certain objectives of engagement, such as informing the public, strengthening trust, and defending science from misinformation, compared to others at their university [36]. Similarly, while more than half of scientists at U.S. land-grant institutions said engagement was “very” or “extremely” important to them personally (53%), to their college dean (55%), and to their university (60%), less than a quarter (23%) thought that engagement was important to their colleagues [7]. This could indicate that perceived importance of engagement to others at the university may be an overlooked measure of institutional culture. Building on this research, we ask the following research question:

RQ2: How do the five dimensions of engagement activities relate to institutional culture, specifically the perceived importance of engagement to the (a) department and the (b) university?

#### Professional position.

Demographic factors related to job requirements and professional position can also impact scientists’ willingness to participate in public engagement. Such factors include academic age, academic division, and whether an individual holds an extension appointment.

Some evidence suggests that scientists’ willingness to participate in public engagement varies by career stage or academic age. For example, one study found that younger scientists were more willing to participate in forms of online engagement, but age had a varied impact on willingness to participate in face-to-face and media engagement activities [20]. Although age was negatively associated with microbiologists’ willingness to participate in both face-to-face and online engagement [22], it did not similarly predict nanoscientists’ intentions to participate in public communication activities [25]. Other work suggests that, compared with their more senior colleagues, early-career scientists are driving the push to participate in public engagement [10,57]. Importantly, willingness to participate in engagement among early-career scientists may not reflect trends in actual engagement participation. For instance, academic age, specifically being a later-career scientist, is associated with increased participation in public scholarship activities [19].

Tenure status and requirements for tenure promotion at U.S. institutions may also affect engagement behavior. Some academics have expressed the view that participating in engagement before achieving tenure is more challenging [10,58]. The goal of attaining tenure can make scientists more “careerist,” meaning they may prioritize the specific requirements to achieve tenure over participating in public engagement activities [59], something which could impact early-career scientists. Recently, researchers have explored how the inclusion of public engagement language in tenure promotion guidelines at land-grant institutions impacts scientists’ participation in public scholarship [19]. While not shown to be a significant predictor of participation, the authors conclude that institutional factors like tenure expectations may contribute to indirect pathways encouraging engagement.

Other factors, such as a scientist’s academic division (e.g., social, biological, or physical science) and whether they hold an extension appointment, may influence their participation in public engagement. While a scientist’s field of study has been shown to impact actual participation in public engagement, research suggests it may similarly affect willingness [20,26]. Other research has shown that physical and life scientists were more willing to participate in informal science education as compared to social scientists, whereas social scientists were significantly more willing to participate in public scholarship [35]. Additionally, many scientists at U.S. land-grant institutions hold extension appointments, which typically require that some percentage of the scientist’s research efforts are directly tied to outreach and engagement with communities throughout their state [60,61]. Building on this work, we examine the following research question:

RQ3: How do the five dimensions of engagement activities relate to professional position, specifically (a) academic age, being in the (b) biological, (c) social, and (d) physical science divisions, and (e) holding an extension appointment?

#### Attitudes towards and perceived goals of engagement.

Scientists may also have different attitudes and engagement goals that influence their participation in public engagement activities. This includes, for example, attitudes related to participation in engagement and personal enjoyment, increased research visibility, the availability of opportunities to engage, and the perceived value of public input.

The belief that public engagement is enjoyable is, unsurprisingly, associated with a higher motivation to engage in those activities that are viewed as enjoyable. One study found, for example, that enjoyment was a key motivator for participation in a science fair, especially among younger scientists [62]. Similarly, a sense of personal satisfaction and enjoyment can be a motivation for faculty to participate in public engagement, broadly [10,32]. Research has also explored the relationship between engagement and increased visibility of research. Work in this space has primarily focused on the influence of engagement with media and social media. For example, a study comparing neuroscientists from the United States and Germany found that about six-in-ten American scientists viewed media visibility as having a “mostly positive impact,” with over a third of German scientists feeling the same [63]. Other work has connected perceptions of media more directly to engagement, such as the development of the ‘presumed media influence index’ by Dudo and colleagues who found perceptions of media influence to be positively correlated with willingness to engage [25]. The promotion of academic research on social media channels such as Twitter (now ‘X’) is also associated with measures of increased visibility such as higher number of citations [64] and increased h-index [65].

Another factor that has been explored is the perceived value of public input as a form of engagement. The role of the public in engagement activities varies across models of engagement [30]. In a 2018 survey of graduate students and science faculty, both groups indicated that it is important to include public input in decision-making about science issues [37]. As previously discussed, some forms of engagement encourage public input – potentially influencing research processes or decision-making. This could, for example, involve working directly with public audiences, which is reflected in our dimension of engagement activities focused on stakeholder-focused engagement. Additionally, scientists’ may be influenced by their perceptions of the availability of opportunities to participate in different types of public engagement activities. Although some types of activities are broadly available (e.g., engagement on social media), others, such as participating in a science festival or presenting research to the public, require infrastructure and therefore opportunity may be limited if those activities are not offered through the scientist’s primary institution.

Finally, researchers have explored a variety of potential goals for scientists’ engagement. Some goals broadly relate to outcomes for the public such as increasing excitement about science [1], educating the public [4], and ensuring that science is valued or appreciated [16]. Other goals may be more directed at specific audiences or tied to specific types of engagement, such as shaping policy [4] or ensuring that policymakers use scientific evidence [16]. Building on this research, we ask the following research question:

RQ4: How do the five dimensions of engagement activities relate to attitudes towards engagement, specifically perceptions of (a) opportunities to participate in engagement, (b) personal satisfaction and enjoyment of engagement activities, (c) the perceived influence engagement on increased visibility of research, (d) the perceived value of public input, and (e) agreement that a goal of engagement is to help shape policy?

In sum, to assess the construct validity for the five dimensions of public engagement activities (public scholarship, educational activities, direct engagement with public audiences, stakeholder-focused collaboration, and industry engagement), we examine how willingness relates to (1) **institutional culture and norms** through scientists’ perceptions of the importance of public engagement to various audiences within their institution, to scientists’ (2) **professional position** including academic age, academic division, and whether the respondent’s faculty position includes an extension appointment, and to scientists’ (3) **attitudes towards engagement** through the influences of personal satisfaction, research visibility, and availability of opportunities to engage, as well as the perceived value of public input and the engagement goal of shaping policy. In addition to these three areas, we also examine how participants’ willingness relates to their past participation in the five dimensions of engagement activities. We propose the following hypothesis:

H1: Past participation in each of the five dimensions of engagement activities will be positively correlated with the corresponding dimensions for willingness to participate in public engagement.

## Methods

To conduct our concept explication and to explore the reliability and validity of the five dimensions of public engagement activities, we fielded a survey with tenure-track science faculty at a large public land-grant university in the United States in the spring of 2021. This study received Institutional Review Board approval for all parts of the data collection and analyses. This study (ID: 2020−0630) was determined to meet the criteria for exempt human subjects by the ED/SBS IRB at the University of Wisconsin–Madison.

### Survey design

The online survey was pre-tested with several members of our research group. It was fielded by the university’s Survey Center and participants were recruited between February 18, 2021, and March 22, 2021. Informed consent was collected electronically through the online survey. Participants were provided a consent form with explanations of the study and possible consequences on the first screen of the survey. They were then asked to indicate whether they consented to participate in the study. Participants who did not consent to participate were immediately terminated from the survey. Those who consented were then taken to the full survey. No incentive for participation was provided. The sample included tenure-track science faculty from the biological, social, and physical science divisions at the university. The final sample (n = 528) had a minimum response rate (RR1) of 27% [66]. The sample is representative of the university’s faculty population regarding gender, race, and academic rank with none of these percentages deviating more than five percentage points from the faculty data of the same year.

### Survey measures

**Willingness to participate in engagement.** Willingness was measured by asking respondents “how willing would you be to participate in the following activities in your professional capacity?” Responses were measured on a 5-point scale from 1 = “not at all willing” to 5 = “extremely willing” and included 13 different engagement activities: (1) publishing non-academic pieces, such as op-eds or popular news articles; (2) participating in interviews with journalists; (3) interacting with government bodies or officials, such as providing expertise to a policymaker or testifying as an expert; (4) participating in science education efforts in K-12 environments; (5) participating in science festivals; (6) interacting with students outside of curricular activities; (7) presenting research to the public, such as at a science café or formal lecture series; (8) engaging on social media about research; (9) discussing research during everyday interactions with the public; (10) including members of the public directly in the research process, such as through citizen science; (11) working on extension-related activities; (12) translating research into marketable products; and (13) collaborating with industry or professional stakeholders on research. The descriptive statistics for each item are reported in Table 1.

**Table 1:** Descriptive statistics for five sub-dimensions of engagement activities.

Item	Mean	Std Dev
**Public scholarship**	**3.67**	**0.90**
1. Publishing non-academic pieces	3.20	1.15
2. Participating in interviews with journalists	3.58	1.06
3. Interacting with government bodies or officials	3.80	1.03
**Educational activities**	**3.67**	**0.88**
4. Participating in science education efforts in K-12 environments	3.38	1.14
5. Participating in a science festival	3.26	1.14
6. Interacting with students outside of curricular activities	3.86	0.99
**Direct engagement with public audiences**	**3.67**	**0.87**
7. Presenting research to the public	3.81	0.97
8. Engaging on social media about your research	2.92	1.34
9. Discussing your research during everyday interactions with the public	3.86	1.00
**Stakeholder-focused collaboration**	**3.00**	**1.05**
10. Including members of the public directly in the research process	2.81	1.25
11. Working on extension-related activities	2.98	1.22
**Industry engagement**	**3.50**	**1.12**
12. Collaborating with industry or professional stakeholders on research	3.58	1.18
13. Translating research into marketable products	2.85	1.30

**Past participation**. Our measure of participation used the same 13 items as our willingness measure. Respondents were asked “thinking about an average year, about how frequently do you participate in the following activities in your professional capacity?” Responses were measured on a 5-point scale, from 1 = “never,” 2 = “once a year,” 3 = “a few times a year,” 4 = “once a month,” and 5 = “a few times a month.” Each item was z-scored to reflect standardized measures of participation in the various engagement activities.

**Perceived importance of engagement.** As previously mentioned, we examine institutional culture and norms by measuring scientists’ perceptions of the importance of public engagement to various audiences within their institution. The perceived importance of public engagement was measured by asking respondents “thinking about your own experiences, how important is public engagement to...” the following groups: your fellow faculty members, your department chair or director, your college dean, your tenure division, and the campus as a whole. Responses were measured on a 5-point scale from 1 = “not at all important” to 5 = “extremely important.” We combined responses into two new variables representing actors at different levels: (1) the department, which includes fellow faculty members and the department chair or director (*M* = 3.08, *SD* = 0.89, *r = *0.67, *p* < 0.001); and (2) the university, which includes the college dean, tenure division, and the campus as a whole (*M* = 3.09, *SD* = 0.78, α = 0.76).

**Influence of opportunities to engage.** Availability of opportunities to engage was measured by asking respondents “How much influence does each of the following have on your decision to engage with the public or not... ‘availability of opportunities to engage with the public at [university name]?” Responses were measured on a 7-point scale from 1 = “strong influence to not engage” to 7 = “strong influence to engage” (*M* = 4.99, *SD* = 1.48).

**Influence of personal satisfaction and enjoyment.** Personal satisfaction from engagement was measured by asking respondents “How much influence does each of the following have on your decision to engage with the public or not... ‘personal satisfaction or enjoyment’?”. Responses were measured on a 7-point scale from 1 = “strong influence to not engage” to 7 = “strong influence to engage” (*M* = 5.83, *SD* = 1.27). It should be noted that this measure reflects the influence of personal satisfaction on decisions to engage, not whether the individual is satisfied by or enjoys engagement.

**Influence of increased visibility of research.** Influence on increased visibility of research was measured by asking respondents “How much influence does each of the following have on your decision to engage with the public or not... ‘increased visibility for my research’?”. Responses were measured on a 7-point scale from 1 = “strong influence to not engage” to 7 = “strong influence to engage” (*M* = 5.4, *SD* = 1.08).

**Perceived value of public input.** To measure the perceived value of public input, we combined three items into an index. Two of the three included items were responses to the statement “I think one of the goals of public engagement is…” to (1) “allow the public to have input on the research we do at [university name]” and to (2) “learn what ethical and societal concerns members of the public have with emerging science.” The third included item asked respondents their agreement on the statement, “The public can bring valuable perspectives to discussions about scientific research.” Responses to these items were measured on a 7-point scale from 1 = “strongly disagree” to 7 = “strongly agree” (*M* = 3.81, *SD* = 0.69, α = 0.67).

**Perceived goal of shaping policy.** The perceived goal of shaping policy was measured by responses to the statement “I think one of the goals of public engagement is to… ‘help shape policy and inform the policymaking process’”. Responses were measured on a 7-point scale from 1 = “strongly disagree” to 7 = “strongly agree” (*M* = 4.37, *SD* = 0.69).

**Position demographics.** The demographic measures used in our analysis include whether the respondent’s faculty position includes an extension appointment (yes = 5.3%), academic age, and academic division. Academic age (*M* = 21.6, *SD* = 11.7) was measured by subtracting the year the respondent said they obtained their highest degree from the year the study was conducted (2021). Academic division includes the biological (43.7%), social (28.7%), and physical (25.5%) sciences.

### Analysis

In Part 1 of our analysis, we explore the reliability of the dimensions of public engagement activities that scientists are willing to participate in (RQ1). There are multiple measures to assess reliability, including homogeneity reliability, which relates to the internal consistency of the items that are included in an index [28,29]. Different indicators of internal consistency include inter-item correlations, Cronbach’s alpha, corrected item-total correlation, and Cronbach’s alpha if an item is deleted [29], which we examine here. We further examine the reliability of our dimensions with a confirmatory factor analysis (CFA). To explore how different external factors relate to scientists’ willingness to participate in engagement, in Part 2 of our analysis we examine the construct validity of the five dimensions of engagement activities [53]. First, we compare the dimensions for willingness to engage with those based on measures of past participation in engagement activities (H1). Then, we compare each dimension against a set of external variables that are theoretically related to evaluate how they align (RQ2–4). The reliability and validity analyses were conducted with SPSS version 28.00. The CFA was conducted using R version 2022.12.0.

## Results

### Part 1: Reliability

#### Reliability of the five dimensions of engagement.

As we previously outlined, the five dimensions of public engagement activities we identified include: public scholarship, educational activities, direct engagement with public audiences, stakeholder-focused collaboration, and industry engagement. The specific activities included in each dimension are detailed in Table 1.

As can be seen in the descriptive statistics reported in Table 1, all of the items included in the five dimensions generally behave similarly [29]. To explore the reliability of these dimensions (RQ1), we examined their internal consistency using Cronbach’s alpha (*α*) for dimensions that included three items and Pearson’s Correlation coefficient (*r*) for dimensions with only two items. The results of the reliability tests are included in Table 2. For public scholarship (*α = *0.77), educational activities (*α = *0.74), and direct engagement with public audiences (*α = *0.68), the Cronbach’s alpha is near or above.70, which is considered an acceptable level of reliability for an index [67]. The results of the Pearson’s correlations for stakeholder-focused collaboration (*r = *0.44) and industry engagement (*r = *0.62) indicate moderate fit [68]. It should be noted, however, that fit thresholds are often subjective and can vary greatly depending on the complexity of concept that is measured [28].

**Table 2:** Results of reliability tests for the five sub-dimensions of engagement activities.

	Corrected Item-Total Correlation	Cronbach’s Alpha if Deleted
**Public scholarship (α = 0.77)**		
1. Publishing non-academic pieces	0.63	0.66
2. Participating in interviews with journalists	0.61	0.69
3. Interacting with government bodies or officials	0.58	0.73
**Educational activities (α = 0.74)**		
4. Participating in science education efforts in K-12 environments	0.67	0.51
5. Participating in a science festival	0.56	0.65
6. Interacting with students outside of curricular activities	0.47	0.75
**Direct engagement with public audiences (α = 0.68)**		
7. Presenting research to the public	0.44	0.69
8. Engaging on social media about your research	0.51	0.57
9. Discussing your research during everyday interactions with the public	0.56	0.50
**Stakeholder-focused collaboration (*r* = 0.44)**		
10. Including members of the public directly in the research process	–	–
11. Working on extension-related activities	–	–
**Industry engagement (*r* = 0.62)**		
12. Collaborating with industry or professional stakeholders on research	–	–
13. Translating research into marketable products	–	–

*Note:* for the indices with three items, we used Cronbach’s Alpha (*α*) and for those with only two items we used Pearson’s Correlation (*r*) as the test of reliability.

#### Confirmatory factor analysis.

We also ran a confirmatory factor analysis (CFA) using the statistical package *lavaan* in *R* to check the existence of the five latent variables corresponding to our five dimensions (see Table 3). We examine three fit statistics to assess our CFA results. The Root Mean Square Error of Approximation (RMSEA) is 0.09, which is above the recommended upper threshold of 0.08 for acceptable model fit [69]. Additionally, the Comparative Fit Index (CFI) and the Tucker Lewis Index (TLI) are below the preferred 0.95 threshold (they are 0.90 and 0.86, respectively) [70]. While these results indicate that the model could be improved, they may be explained in part by non-random measurement error [52] resulting from similarities in survey item question wording and the items appearing close together in the survey. In cases where measures come from similarly worded test items like the 13 items we use that represent different aspects of a similar concept, the specification of correlated measurement errors may be justified [71]. In other words, while we expect to see relationships between items based on their shared influence of the latent dimension (factor), covariation between them may occur because of other sources [71], representing non-random measurement error.

**Table 3:** Willingness to participate CFA factor loadings for the standardized solution.

Item	SD	Low CI	Up CI	P-value
**Public scholarship**				
1. Publishing non-academic pieces	0.79	0.74	0.84	0.00
2. Participating in interviews with journalists	0.73	0.68	0.79	0.00
3. Interacting with government bodies or officials	0.68	0.62	0.74	0.00
**Educational activities**				
4. Participating in science education efforts in K-12 environments	0.78	0.73	0.84	0.00
5. Participating in a science festival	0.73	0.67	0.79	0.00
6. Interacting with students outside of curricular activities	0.60	0.53	0.67	0.00
**Direct engagement with public audiences**				
7. Presenting research to the public	0.78	0.73	0.83	0.00
8. Engaging on social media about your research	0.53	0.46	0.60	0.00
9. Discussing your research during everyday interactions with the public	0.67	0.62	0.73	0.00
**Stakeholder-focused collaboration**				
10. Including members of the public directly in the research process	0.66	0.59	0.74	0.00
11. Working on extension-related activities	0.67	0.59	0.74	0.00
**Industry engagement**				
12. Collaborating with industry or professional stakeholders on research	0.64	0.55	0.74	0.00
13. Translating research into marketable products	0.97	0.85	1.08	0.00

*Note:* reported values include the estimated standardized factor loading (SD), the lower and upper confidence intervals for the factor loading (Low CI and Up CI), and the p-value.

To explore this further, we examined the correlated measurement errors with a matrix showing the correlations of the indicator error terms. By doing so, we were able to confirm that there are several correlations between item errors that are greater than |.10|, indicating a likely specification error [72]. The correlations that exceed this threshold appear across the five dimensions public engagement activities (see S1 Table and S2 Table). As noted above, this is not surprising given similarities in item wording and because items appear in the same battery of the survey. Overall, this correlated error matrix helps us further explain why the five dimensions we defined are not strongly supported by the CFA findings. Despite this, there are many reasons why these dimensions make sense given the activity types measured, which we examine in Part 2 by evaluating the construct validity of the dimensions.

### Part 2: Construct validity

#### Comparisons with past engagement participation.

As noted above, our survey included a battery of questions asking scientists about their past participation in the same 13 types of engagement activities. As one measure of validation, we compared willingness to participate in engagement with the corresponding dimensions for past participation in engagement. The results of the reliability tests for the five dimensions of participation are included in Table 4. In support of H1, the correlations between the dimensions of public engagement activities for willingness and past participation show that each dimension is most highly correlated with its parallel dimension (Table 5). The correlations suggest that while willingness and participation are closely related, they are measuring separate concepts. We also ran a CFA using the participation variables and the results are like our CFA with willingness to participate. The findings from the CFA show an RMSEA of 0.07, which indicates acceptable model fit [69]. The CFI and TLI are slightly below the ideal 0.95 threshold (0.92 and 0.89, respectively) [70].

**Table 4:** Reliability for the five sub-dimensions of past participation in engagement.

	Corrected Item-Total Correlation	Cronbach’s Alpha if Deleted
**Public scholarship (α = 0.77)**		
1. Publishing non-academic pieces	0.61	0.69
2. Participating in interviews with journalists	0.64	0.69
3. Interacting with government bodies or officials	0.58	0.73
**Educational activities (α = 0.56)**		
4. Participating in science education efforts in K-12 environments	0.46	0.32
5. Participating in a science festival	0.40	0.42
6. Interacting with students outside of curricular activities	0.27	0.62
**Direct engagement with public audiences (α = 0.63)**		
7. Presenting research to the public	0.51	0.42
8. Engaging on social media about your research	0.37	0.61
9. Discussing your research during everyday interactions with the public	0.43	0.54
**Stakeholder-focused collaboration (*r* = 0.33)**		
10. Including members of the public directly in the research process	–	–
11. Working on extension-related activities	–	–
**Industry engagement (*r* = 0.47)**		
12. Collaborating with industry or professional stakeholders on research	–	–
13. Translating research into marketable products	–	–

*Note:* for the indices with three items, we used Cronbach’s Alpha (*α*) and for those with only two items we used Pearson’s Correlation (*r*) as the test of reliability.

**Table 5:** Pearson correlations between willingness to participate in engagement and past participation in engagement.

		Past participation									
		Public scholarship		Educational activities		Direct engagement with public		Stakeholder-focused collaboration		Industry engagement	
		*r*	*p*	*r*	*p*	*r*	*p*	*r*	*p*	*r*	*p*
**Willingness**	Public scholarship	**0.55**	<.001	0.23	<.001	0.48	<.001	0.30	<.001	0.12	0.010
	Educational activities	0.03	0.540	**0.54**	<.001	0.24	<.001	0.09	0.053	−0.06	0.164
	Direct engagement with public	0.36	<.001	0.27	<.001	**0.68**	<.001	0.22	<.001	0.05	0.254
	Stakeholder-focused collaboration	0.29	<.001	0.22	<.001	0.35	<.001	**0.62**	<.001	0.21	<.001
	Industry engagement	0.15	0.001	0.06	0.162	0.12	0.011	0.21	<.001	**0.60**	<.001

*Note:* reported values include Pearson’s Correlation coefficient (*r*) and the p-value (*p*).

#### Comparisons with external variables.

To assess the construct validity of our five dimensions of engagement [53], we examined the association of each dimension with nine external variables that have been previously shown to impact willingness to engage. These include perceived importance of institutional culture (RQ2a-b), academic age (RQ3a), academic division (RQ3b-d), whether the scientist holds an extension appointment (RQ3e), perceptions of opportunities to engage at the scientists’ institution (RQ4a), personal satisfaction and enjoyment of engagement activities (RQ4b), the perceived influence engagement on increased visibility of research (RQ4c), the perceived value of public input (RQ4d), and agreement that a goal of engagement is to help shape policy (RQ4e). The correlations of the external variables with each of our five dimensions are discussed below and are detailed in Table 6.

**Table 6:** Pearson correlations of willingness to participate dimensions with external variables.

	Public scholarship		Educational activities		Direct engagement with public		Stakeholder-focused collaboration		Industry engagement	
	*r*	*p*	*r*	*p*	*r*	*p*	*r*	*p*	*r*	*p*
Biological Science^a^	−0.15	<.001	0.00	0.986	−0.03	0.481	−0.02	0.747	0.09	0.040
Physical Science^a^	−0.16	<.001	0.01	0.785	−0.18	<.001	−0.15	<.001	0.07	0.115
Social Science^a^	0.30	<.001	−0.01	0.778	0.18	<.001	0.16	<.001	−0.17	<.001
Academic age	−0.04	0.357	−0.13	0.006	−0.19	<.001	−0.10	0.028	−0.06	0.239
Extension^a^	0.14	0.002	−0.09	0.042	0.08	0.081	0.32	<.001	0.14	0.002
Importance to department	0.20	<.001	0.23	<.001	0.18	<.001	0.24	<.001	0.06	0.176
Importance to university	0.05	0.313	0.15	<.001	0.09	0.063	0.06	0.194	0.11	0.012
Influence of opportunities	0.21	<.001	0.21	<.001	0.28	<.001	0.20	<.001	0.04	0.395
Influence of satisfaction	0.45	<.001	0.33	<.001	0.42	<.001	0.32	<.001	0.12	0.008
Influence of visibility	0.32	<.001	0.03	0.495	0.34	<.001	0.24	<.001	0.24	<.001
Value of public input	0.16	<.001	0.17	<.001	0.23	<.001	0.41	<.001	0.17	<.001
Goal to shape policy	0.21	<.001	0.07	0.13	0.15	<.001	0.17	<.001	0.05	0.284

*Note:* reported values include Pearson’s Correlation coefficient (*r*) and the p-value (*p*). ^a^For our dichotomous variables, biological, physical, and social science academic divisions and extension appointment, correlations reflect Point-Biserial Correlations (*r*_*pb*_).

**Public scholarship.** Whereas willingness to participate in public scholarship is positively correlated with being in the social sciences (*r*_*pb*_ = 0.30, *p* < .001), it is negatively correlated with the biological and physical science divisions (*r*_*pb*_ = −0.15 and *r*_*pb*_ = −0.16, *p* < .001, respectively). Public scholarship is positively correlated with the perceived influence of opportunities for engagement (*r* = 0.21, *p* < .001), personal enjoyment and satisfaction (*r* = 0.45, *p* < .001), and increased visibility for research (*r* = 0.32, *p* < .001). It is also positively correlated with holding an extension appointment (*r*_*pb*_ = 0.14, *p* = .002), the value of public input (*r* = 0.16, *p* < .001), and perceived importance to the department (*r* = 0.20, *p* < .001). Willingness to participate in public scholarship is positively correlated with the belief that one of the goals of public engagement is shaping public policy (*r* = 0.21, *p* < .001). There are no correlations with academic age or perceived importance to the university.

**Educational activities.** Willingness to participate in educational activities is negatively correlated with holding an extension appointment (*r*_*pb *_= −0.09, *p* = .042) and academic age (*r* = −0.13, *p* = .006). It is positively correlated with the perceived importance of engagement to both the department (*r* = 0.23, *p* < .001) and university (*r* = 0.15, *p* < .001). Willingness to participate in educational activities is also positively correlated with the perceived influence of opportunities for engagement (*r* = 0.21, *p* < .001) and personal enjoyment and satisfaction (*r* = 0.33, *p* < .001), as well as with the value of public input (*r* = 0.17, *p* < .001). There are no correlations with the influence of increased visibility, the perception that a goal of public engagement is to help shape policy, or academic division.

**Direct engagement with public audiences.** There are directionally opposite correlations between being in the physical and social sciences division for willingness to participate in direct engagement with public audiences (*r*_*pb *_= −0.18 vs. *r*_*pb *_= 0.18, *p* < .001, respectively). This dimension is negatively correlated with academic age (*r* = −0.19 *p* < .001). Direct engagement with public audiences is positively correlated with the perceived influence of opportunities for engagement (*r* = 0.28, *p* < .001), personal enjoyment and satisfaction (*r* = 0.42, *p* < .001), and increased visibility for research (*r* = 0.34, *p* < .001). It is also positively correlated with the perceived importance to the department (*r* = 0.18, *p* < .001), the value of public input (*r* = 0.23 *p* < .001), and the perception that a goal of public engagement is to help shape policy (*r* = 0.15 *p* < .001). There are no significant correlations between willingness to participate in direct engagement with public audiences and being in the biological sciences division, holding an extension appointment, or perceived importance to the university.

**Stakeholder-focused collaboration.** There are directionally opposite correlations for academic division and willingness to participate in stakeholder-focused collaboration. While being in the physical sciences is negatively correlated (*r*_*pb *_= −0.15, *p* < .001), being in the social sciences division has a positive correlation (*r*_*pb*_ = 0.16, *p* < .001). It is negatively correlated with academic age (*r* = −0.10, *p* = .028). Our findings show that holding an extension appointment is more highly correlated with stakeholder-focused collaboration (*r*_*pb*_ = 0.32, *p* < .001) than any of the other dimensions of engagement. The same is true for the perceived value of public input (*r* = 0.41, *p* < .001). Willingness to participate in stakeholder-focused collaboration is positively correlated with the perceived influence of opportunities for engagement (*r* = 0.20, *p* < .001), personal enjoyment and satisfaction (*r* = 0.32, *p* < .001), and increased visibility for research (*r* = 0.24, *p* < .001). It is also positively correlated with the perceived importance to the department (*r* = 0.24, *p* < .001) and the perception that a goal of public engagement is to help shape policy (*r* = 0.17, *p* < .001). There are no correlations with being in the biological sciences division or perceived importance to the university.

**Industry engagement**. Willingness to participate in industry engagement is the only dimension that is positively correlated with being in the biological sciences division (*r*_*pb*_ = 0.09, *p* = 0.040). Additionally, it is the only dimension to have a negative correlation with being in social sciences (*r*_*pb*_ = −0.17, *p* < .001). It is positively correlated with holding an extension appointment (*r*_*pb*_ = 0.14, *p* = .002). Willingness to participate in industry engagement is correlated with perceived importance of engagement to the university (*r = *0.11, *p* = .012), but not to department, in contrast with other dimensions of engagement. It is positively correlated with the visibility of research (*r* = 0.24, *p* < .001). While it is also positively correlated with the perceived influence of personal satisfaction and enjoyment (*r* = 0.12, *p* = .008), the correlation is lower compared with the other dimensions. There is a positive correlation with the value of public input (*r* = 0.17, *p* < .001). There are no correlations between industry engagement and being in the physical sciences division, opportunities to engage at the university, perceived importance to the department, and the perception that a goal of engagement is to help shape policy.

## Discussion

This study offers one of the first empirical explications of the concept of scientists’ willingness to participate in engagement, identifying five dimensions of public engagement activities: public scholarship, educational activities, direct engagement with public audiences, stakeholder-focused collaboration, and industry engagement. Below we discuss the findings from our reliability and validity testing that support this categorization and examine trends and differences across the dimensions.

Before turning to our discussion, it is important to note a few data-related considerations. First, our sample is restricted to perspectives of tenure-track science faculty at a single land-grant university in the United States. Having said this, previous research on public engagement at U.S. land-grant institutions found minimal variation across universities regarding scientists’ public engagement participation [19]. In other words, while our study is limited to a single university context, it may map reasonably well onto science faculty perspectives at other U.S. land-grant institutions. Regardless, future research should explore these questions for other institutions and at other types of research organizations in the United States. Additionally, only including tenure-track scientists may leave some scientists out of the sample [73], which is something future work could account for.

Second are considerations of measurement error. Because the items included in our dimensions of engagement are not necessarily mutually exclusive, there is a chance that we introduced non-random measurement error [52], which reduces our ability to distinguish between dimensions and creates some measurement overlap. One way to reduce that measurement error would be to develop more specific items representing a broader range of engagement activities. However, because we have a heterogeneous sample, there is a risk that participants might not see themselves in very specific items that would be more distinct. Exploring the use of additional activities is another direction for future research in this space, although it will be important to balance this with considerations about using a parsimonious survey scale.

Ultimately, the five dimensions of public engagement activities that we identified are supported by our reliability testing (RQ1). As previously discussed, some of the discrepancies with our CFA are likely due to measurement error and should be considered in future studies that further refine how to measure these dimensions. We define each of the dimensions of engagement activities below:

1. **Public scholarship** refers to scientists using their expertise to inform public debate, discourse, and democratic decision-making related to scientific development and science policy, often with targeted audiences such as policymakers and the media. This may include activities such as writing opinion editorials or participating in interviews with journalists, providing public testimony, and participating in public forums.
2. **Educational activities** refer to interactions with students or other publics facilitated through an educational institution that are outside of the scope of any formal teaching responsibilities that a scientist may have. These can include activities such as participation in science festivals or in K-12 environments, where learning is a target outcome.
3. **Direct engagement with public audiences** includes interactions about science issues in both organized and spontaneous formats such as engagement on social media or participation in a science café (without an explicit goal or modality focused on learning outcomes). These types of activities involve direct engagement with public audiences and may be initiated by scientists outside of any formal institutional structures.
4. **Stakeholder-focused collaboration** involves stakeholders or members of the public in a more collaborative effort, such as participation and input into research projects. Other examples include participation in extension-related projects.
5. **Industry engagement** refers to interactions with non-academic, private organizations or stakeholders such as translating research into marketable products or collaborations with industry.

Our validity testing (RQ2–4) provides more context for each of these dimensions by demonstrating how they correlate with relevant external variables.

We examined the role of institutional culture on willingness through measures of the perceived importance of engagement to the department (faculty and departmental head) and the university (college dean, tenure division, campus as a whole). The perception that public engagement is important to the scientists’ department is significantly positively correlated with willingness to engage in four of the five dimensions we examined, except for industry engagement (RQ2a). On the other hand, the perception that public engagement is important to the scientists’ university is only correlated with educational activities (RQ2b). Although prior research on engagement and institutional culture has been somewhat mixed [e.g., 19,20], our findings underscore the potential influence of institutional culture on scientists’ willingness to participate in public engagement. They also suggest that a focus on institutional culture at the departmental level may be more broadly relevant to participation in a variety of engagement activities.

Next, we looked at how three factors related to scientists’ professional position—academic age, academic division, and whether their position includes an extension appointment—correspond with each of the dimensions. We found that academic age, or the number of years since a scientist completed their highest degree, is negatively correlated with willingness to participate in educational activities, direct engagement with public audiences, and stakeholder-focused collaboration (RQ3a). Our findings support the idea that more junior academics are more willing to participate in some types of engagement [10,20]. However, this does not necessarily translate into actual participation in engagement activities, which, for some forms of engagement, is higher among those of greater academic age [19]. This association between academic age and willingness could be due in part to expectations around institutional tenure promotion requirements, which may or may not recognize participation in certain public engagement activities [19]. This may be especially relevant for more junior faculty who are non-tenured and view tenure requirements as a barrier to participating in engagement [10]. It is important to keep in mind, however, that the mean academic age for our sample was 21.6 years, which represents the perspectives of more senior faculty. Future studies should explore the nuances of these differences in academic age and their relationship to willingness.

Our findings show differences in attitudes towards engagement depending on which academic division a scientist belongs to. Being in the social sciences is correlated with four of the five engagement dimensions, except for educational activities which shows no correlations by division (RQ3c). Consistent with previous research, we found that being in the social sciences was positively correlated with willingness to participate in public scholarship [35]. This could be explained, in part, by the fact that social scientists tend to have more experience with news media than other science fields [63]. Similarly, correlations with direct engagement could be due to a growing use of social media for online scholarly engagement within fields such as communication [74]. The relationship between social science and willingness to participate in stakeholder-focused activities is also in line with recent calls for community-engaged collaborations by researchers in fields such as sociology [75] and science communication [76]. In contrast to the social sciences, being in the biological (RQ3b) or physical (RQ3d) science divisions is either negatively or not correlated with the five dimensions, with one exception. Industry engagement is negatively correlated with being a social scientist and positively correlated with being a biological scientist. This difference makes sense given that being in a biological science field may be more closely aligned with industry than fields in the social sciences. Overall, our findings are in line with previous research that suggests field-specific differences in willingness to participate in engagement [e.g., 20,26].

Since faculty with extension appointments are expected to incorporate various publics into research activities [60], it would make sense that those who hold an extension appointment would be more willing to participate in stakeholder-focused collaboration, which was supported by our findings (RQ3e). At the same time, although not as highly correlated, we also see relationships between holding an extension appointment and willingness to participate in both public scholarship and industry engagement. For sciences that have close relationships with industry partners (e.g., agricultural sciences), extension appointments may be more directly tied to those industry relationships. Holding an extension appointment may also result in more visibility and thus potentially more opportunities to engage with the media or policymakers (i.e., public scholarship activities).

Additionally, we explored how the influence of three factors—perceived satisfaction or enjoyment from engagement, availability of opportunities to engage, and the increased visibility of research—correlated across our dimensions. For all five dimensions, our results show significant positive relationships with the influence of personal satisfaction or enjoyment (RQ4b). It makes sense that feeling a sense of satisfaction or enjoyment from public engagement would relate to willingness to engage – those who like engagement are likely the ones who would choose to participate in engagement. This aligns with studies that have identified enjoyment as a motivating factor for faculty participation in public engagement, broadly [10,32]. Compared with the other dimensions, industry engagement is less strongly correlated with the perceived influence of personal satisfaction and enjoyment. It possible that industry engagement is more closely tied to professional expectations and responsibilities, and therefore less influenced by personal satisfaction. However, as noted above, industry engagement was not correlated with perceived importance of public engagement to the scientists’ department or university. A similar pattern can be seen with the influence of the availability of opportunities to engage, which is positively correlated with all dimensions except for industry engagement (RQ4a). Although scientists recognize industry as an audience for engagement [15], additional research is needed to explore the motivating factors associated with participation in industry engagement activities. Finally, the perception that engagement will have an influence on increasing visibility of research is positively and significantly correlated with all dimensions except for educational activities (RQ4c). It is possible that because educational activities have learning as a target outcome, there is less of an emphasis on increasing research visibility through those types of activities.

Furthermore, while all dimensions are positively correlated with the perceived value of public input (RQ4d), the correlation is the strongest for stakeholder-focused collaboration. Scientists who agree that public perspectives are valuable in discussions about scientific research are likely to be more willing to participate in various engagement activities that directly involve publics in research collaborations. The stronger correlation with participation in stakeholder-focused collaboration is reasonable given that those activities are intended to incorporate public input directly into the research process (e.g., through extension-related work).

Finally, the belief that a goal of public engagement is to shape policy is correlated with three of five dimensions: public scholarship, direct engagement with the public, and stakeholder-focused collaboration (RQ4e). It makes sense that we would see this relationship with public scholarship since this measure includes providing expertise to a policymaker or testifying as an expert. Similarly, direct engagement with the public and stakeholder-focused collaboration can involve a variety of individuals and groups, which may very well include policy-related entities or in relation to specific policy issues. In contrast, it may be that the policymaking process is less relevant to the audiences involved in educational activities and industry engagement, potentially explaining the lack of correlations.

The current study provides a robust categorization of willingness to engage that can serve as a blueprint for future research in this space. Overall, the five dimensions we have identified represent distinct measures of willingness to engage that can be standardized in follow-up studies. In addition to providing a more general measure of the activities that scientists are willing to engage in, future research could examine how discipline and or field specific activities may align with these different dimensions of engagement. Building on the current study, future research might also explore how other institutional factors, such as participation in public engagement [77] and science communication [19,21] training programs, influence willingness to participate in these different engagement activities. To better understand how to navigate the engagement expectations that scientists face, we need empirical data that allows for the examination of engagement trends among the scientific community. This is increasingly important advances in science and technology underscore the need for scientists’ engagement with the public.

## Supporting information

S1 Table Correlation matrix of the residual (indicator) error terms for public engagement activities. (DOCX)

S2 Table Covariance matrix of the residual (indicator) error terms for public engagement activities. (DOCX)
