# Supplementary material for: University scientists’ willingness to participate in public engagement: A concept explication
Source: PLoS One. 2025 Nov 25;20(11):e0337189. doi: 10.1371/journal.pone.0337189 (PMC12646421; doi:10.1371/journal.pone.0337189)
Supplement: S2 Table — (DOCX) [file pone.0337189.s002.docx]

**University scientists’ willingness to participate in public engagement: A concept explication**

SUPPLEMENTARY MATERIALS

**S2 Table**. Covariance matrix of the residual (indicator) error terms for public engagement activities

|  | Publ. | Int. | Gov | Edu. | Fest. | Stud. | Pres. | Soc. | Disc. | Proc. | Ext. | Prod. | Coll. |
| --- | --- | --- | --- | --- | --- | --- | --- | --- | --- | --- | --- | --- | --- |
| Publish | 0.00 |  |  |  |  |  |  |  |  |  |  |  |  |
| Interview | 0.01 | 0.00 |  |  |  |  |  |  |  |  |  |  |  |
| Gov | -0.01 | 0.01 | 0.00 |  |  |  |  |  |  |  |  |  |  |
| Educate | 0.01 | -0.05 | -0.16 | 0.00 |  |  |  |  |  |  |  |  |  |
| Festival | -0.04 | -0.02 | -0.11 | 0.05 | 0.00 |  |  |  |  |  |  |  |  |
| Students | 0.23 | 0.14 | 0.12 | 0.02 | -0.11 | 0.00 |  |  |  |  |  |  |  |
| Present | 0.00 | 0.01 | -0.02 | -0.07 | 0.01 | 0.08 | 0.00 |  |  |  |  |  |  |
| Social | 0.09 | 0.10 | 0.01 | -0.17 | -0.01 | 0.01 | 0.00 | 0.00 |  |  |  |  |  |
| Discuss | -0.03 | -0.02 | -0.05 | -0.03 | 0.05 | 0.18 | 0.00 | -0.01 | 0.00 |  |  |  |  |
| Process | 0.04 | -0.11 | -0.08 | -0.09 | 0.05 | 0.09 | 0.03 | 0.06 | 0.11 | 0.00 |  |  |  |
| Extension | 0.08 | -0.03 | 0.05 | -0.05 | -0.04 | 0.14 | -0.00 | -0.21 | -0.08 | 0.00 | 0.00 |  |  |
| Product | -0.17 | -0.05 | 0.09 | -0.05 | 0.19 | 0.02 | -0.06 | 0.20 | -0.00 | 0.03 | 0.07 | 0.00 |  |
| Collab | -0.07 | -0.04 | 0.17 | -0.07 | 0.03 | 0.08 | -0.03 | 0.06 | 0.02 | -0.04 | 0.03 | 0.00 | 0.00 |
